# Evaluating the Efficiency of Magnetic Treatment for Feed Water in Reverse Osmosis Processes

**DOI:** 10.3390/membranes13070641

**Published:** 2023-07-01

**Authors:** Qian Lei, Ezinwa Elele, Yueyang Shen, John Tang, Katherine L. Guerra, Frank Leitz, Boris Khusid

**Affiliations:** 1 Department of Chemical and Materials Engineering, New Jersey Institute of Technology, University Heights, Newark, NJ 07102, USA; qian.lei@njit.edu (Q.L.); eoe4@njit.edu (E.E.); shen@njit.edu (Y.S.); jt97@njit.edu (J.T.); 2 Water Treatment Group, Technical Service Center, U.S. Bureau of Reclamation, P.O. Box 25007, Denver, CO 80225, USA; kguerra29@gmail.com (K.L.G.);

**Keywords:** reverse osmosis, mineral scaling, testing methodology, magnetic treatment, scale deposition and morphology

## Abstract

The paper presents a new methodology for short-term (5–25 min) benchtop tests to evaluate the effectiveness of magnetic treatment of feed water for reducing mineral scaling on a reverse osmosis (RO) membrane. Scale deposition is measured at a controlled level of salt supersaturation in water flowing through an RO unit in once-through mode. A magnetic water conditioner is tested in a transient flow regime when variations of the permeate flux along the flow path are insignificant. Scale formation under these conditions is governed by salt crystallization on the membrane surface. The proposed method was implemented to investigate the influence of magnetic treatment on gypsum deposition on RO membranes in supersaturated aqueous CaSO_4_/NaCl solutions. The effects of magnetic water treatment on scale formation under our experimental conditions were found to be statistically insignificant with a confidence level of 95%. However, this outcome should not be considered to negate the potential efficiency of magnetic water treatment in specific applications. The proposed methodology of testing under a controlled level of salt supersaturation will also be useful for evaluating the efficiency of other water treatment technologies.

## 1. Introduction

Reverse osmosis (RO) technology in both industrial processes and the production of potable water has advanced significantly in the past decade, owing to the development of more robust membranes and efficient energy recovery methods [1,2]. However, membrane fouling remains one of the major RO challenges. Scale forms on a membrane when the amount of a sparingly soluble salt in water flowing through an RO system exceeds the salt solubility. Sparingly soluble salts in feed water can crystallize directly onto the RO membrane surface, forming an adherent mineral scale that causes the permeate flux to decline and eventually damages the membrane [3,4,5,6]. The most widespread strategy currently employed to reduce membrane fouling is the use of antiscalant additives in the feed water, which inhibit the nucleation and growth of scale deposits [3,4,7]. Unfortunately, these chemicals, though efficient, may substantially affect the composition of drinking water, making it unsuitable for human consumption [8,9]. Moreover, chemical treatments are highly specific to a particular composition of the feed water with respect to their effectiveness and can be prohibitively expensive for large-scale processes [10]. 

There are a host of currently marketed technologies for anti-scaling and anti-fouling treatment of feed water by passing it through a magnetic or electromagnetic field as a non-chemical alternative to water treatment. Magnetic and electromagnetic conditioners of feed water are commercially available in a wide variety of configurations and can be easily plumbed in or clamped on. These technologies are therefore compelling due to their simplicity and relatively low cost. A majority of marketed devices use an array of permanent magnets, as they are simpler to fabricate and easier to use. Comprehensive reviews of the literature on water treatment in magnetic and electromagnetic softeners are given in Refs. [11,12,13,14,15,16,17,18,19,20,21,22,23] consider this topic from different viewpoints. Whereas there are some inconsistencies between reported results, 86% of studies on membrane systems state that the magnetic water treatment facilitated the reduction of scale deposition [22]. Furthermore, the influence of magnetic treatment could last for many hours after a field has ceased [24]. The potential economic savings of the use of magnetic and electromagnetic water treatment are discussed in detail in [19,22]. 

The number of papers on magnetic and electromagnetic conditioners has risen exponentially, from about 4000 published in the last century to over 3500 published in this century [22]. Two mechanisms are invoked in the literature to explain the efficiency of magnetic treatment on salt solutions [19,20,21,22,24,25]. One of them is attributed to magnetically induced changes in the hydrating water structure around the ions and hydrophobic solid surfaces. The other is that local electrical fields generated in electrically conducting water flowing through a magnetic field influence the motion of ions and charged particles. In both cases, it is agreed that magnetic treatment facilitates bulk precipitation of sparingly soluble salts in feed water. As these particles are carried away by the water flow, the formation of a tightly adherent scale layer is thereby suppressed. Publications reviewed in [11,12,13,14,15,16,17,18,19,20,21,22,23] clearly indicate that the benefits of magnetic treatment of feed water depend strongly on various operating parameters: field strength, exposure time, temperature, channel geometry and material, flow rate, water composition, pH level, type of scale, etc. The treatment effect in membrane processes is expressed in terms of relative changes in the scale deposit and/or permeate flux and/or salt rejection [22]. The treatment improvement is then presented as a function of operating parameters. The reported results vary significantly from one testing setup to another and from one water composition to another, e.g., [11,12,13,17,20,22]. Accordingly, there is currently no agreement on operating parameters for which magnetic water treatment could be efficient in an industrial RO system.

Given their widespread availability, inexpensive magnetic and electromagnetic water conditioners will continue to draw interest across the industry to explore their efficiency in industrial RO systems. For example, the effectiveness of magnetic and electrical water conditioners was tested on feed water to the U.S. Bureau of Reclamation’s 72-million gallon-per-day Yuma Desalting Plant located in Yuma, AZ [26]. Measurements were carried out over several months on an RO pilot unit operating in circulation mode. Both conditioners were found to be ineffective in reducing calcium sulfate scaling. Due to the high cost of industrial tests, there is an urgent need to develop a method to evaluate the chances of success of the testing effort. The critical question is: *How can we design short-term laboratory-scale experiments to explore the ability of a magnetic water conditioner to improve the efficiency of a large industrial RO system?* The paper demonstrates a new methodology disclosed in our patent [27] to address this question.

As scale forms when the salt concentration in water exceeds its solubility, the main idea of [27] is to evaluate the magnetic treatment efficiency at a controlled level of salt supersaturation in feed water. To the best of our knowledge, the effects of magnetic water treatment on scale formation have never been studied at fixed values of salt supersaturation. The approach [27] is based on a certain analogy between precipitation processes in a stirred reactor and in a flow system. The test configuration and experimental procedures described in [27] allow a direct comparison of scale deposits formed in the treated and untreated feed water at the same level of salt supersaturation. This methodology paves the way to use laboratory measurements to predict the efficiency of magnetic water treatment in an industrial RO system. The first step is to measure variations in the water’s electrical conductivity along the flow path through the system of interest. This dataset is taken to determine the range of local water supersaturation in this system by using measurements in a stirred reactor. Tests in a laboratory RO unit are then conducted over this range of local water supersaturation. The magnetic treatment of feed water in this industrial system would be successful only if laboratory testing demonstrated its efficiency.

The methodology [27] was implemented in this paper to investigate the effect of magnetic treatment on gypsum deposition in a supersaturated aqueous CaSO_4_/NaCl solution flowing through a benchtop RO unit. Polarized microscopy with image processing, scanning electron microscopy (SEM), X-ray diffractometry (XRD), and differential scanning calorimetry (DSC) were used to characterize the gypsum scale formed on RO membranes. We consider that the methodology [27] for short-term benchtop tests will also be useful to evaluate the efficiency of other technologies for water treatment in flow systems.

## 2. Materials and Methods

### 2.1. Testing Methodology

Transfer of water through the RO membrane creates strong solute supersaturation in a layer at the membrane surface, referred to as concentration polarization [28]. During the transient regime, scale formation on the membrane surface consists of two main steps: nucleation and growth of salt crystals [29]. Nucleation processes in a supersaturated solution when it flows in a channel and when it is agitated in a stirred vessel might differ. However, once initial nuclei are formed, processes of crystal growth in the surrounding supersaturated solution in a flow system and in a stirred vessel have much in common as they are governed by similar mechanisms.

The proposed way to characterize the precipitation kinetics in a supersaturated solution in a stirred vessel is to record changes with time in the electrical conductivity, the intensity of scattered and transmitted light, or the concentration of specific ions (e.g., Ca^2+^ ions for gypsum) [27]. The solution supersaturation is then characterized by the parameter $\xi \left(t\right)$. The value of ξ is calculated as the ratio between a change in one of these characteristics during a period of time t and the total change. Accordingly, ξ=0 and ξ=1 are the values at the beginning and the end of the precipitation process, respectively. Therefore, the first step in our test was to measure $\xi \left(t\right)$ in a stirred reactor by recording changes in the three parameters: electrical conductivity, turbidity, and concentration of Ca^2+^ ions. A dataset on $\xi \left(t\right)$ was then used to compute the value of ξ for the RO unit from measurements of the solution electrical conductivity at the RO inlet and outlet.

**Figure 1:**
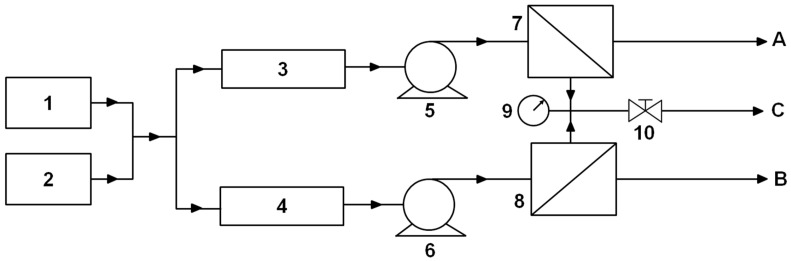
Flow diagram [27]: A, permeate from treated feed; B, permeate from untreated feed; C, retentate. 1, CaCl_2_/NaCl solution; 2, Na_2_SO_4_/NaCl solution; 3, magnetic treatment unit; 4, dummy unit; 5, 6, constant flow rate pumps; 7, 8, RO units; 9, pressure gauge; 10, needle valve. The hardware described in Section 2.3.2.

To maintain the same degree of solution supersaturation flowing through the RO unit, experiments were carried out in a single-pass mode of flow (Figure 1).

To simulate the effects of concentration polarization creating high solute supersaturation at the RO membrane surface, experiments were conducted to determine the kinetic regime of scale growth when it is controlled by surface crystallization. Under these experimental conditions, the percentage of the membrane surface area covered with scale deposits did not change along and across the flow. To improve the resolution of the magnetic treatment effects, we adopted a differential scheme by using two similar branches, A and B, operating at the same flow rate and transmembrane pressure (Figure 1). Both branches were fed with the same supersaturated solution. Each branch included a pump and an RO unit. One needle valve was used to regulate flow rates in both branches. The RO unit in branch A received the solution that passed through the magnetic field. The RO unit in branch B received the solution that passed through a similar flow assembly (referred to as the dummy unit) but was not exposed to the field (Figure 1).

### 2.2. Materials

All experiments were carried out on aqueous solutions of CaCl_2_, NaCl, and Na_2_SO_4_ that were prepared from ACS-grade (>99%) powders of (NaCl), calcium chloride dihydrate (CaCl_2_·2H_2_O), and sodium sulfate (Na_2_SO_4_) acquired from Millipore Sigma (St. Louis, MO, USA). Stock solutions of CaCl_2_/NaCl and Na_2_SO_4_/NaCl were prepared in 5 L tanks by adding appropriate molar amounts of NaCl, CaCl_2_·2H_2_O, and Na_2_SO_4_ to de-ionized (DI) water. A proper amount of salt powder was weighed on a Mettler Toledo AL204 Laboratory Balance (Mettler Toledo, Columbus, OH, USA). Stock solutions were allowed to stand to ensure complete dissolution of salts and were then filtered through a Supor 200 PES Membrane Disc Filter with 0.2 µm pores (Pall, Cortland, NY, USA) to remove remaining particulates. An aqueous solution supersaturated with CaSO_4_ was formed by mixing two stock solutions at the desired values of x and y:

(xNaCl + yCaCl2) + (xNaCl+ yNa2SO4) → yCaSO4+ 2(x + y)NaCl(1)

A supersaturated solution is conventionally characterized by the supersaturation ratio $S_{g}$ that is considered with respect to the equilibrium between ${Ca}^{2+}$ and $SO_{4}^{2-}$ ions in the solution and in the gypsum crystal:

$$CaSO_{4}\cdot 2H_{2}O⇆{Ca}^{2+}+SO_{4}^{2-}+2H_{2}O$$

so that

$$S_{g}=a_{{Ca}^{2+}}a_{SO_{4}^{2-}}a_{H_{2}O}^{2}/{\left(a_{{Ca}^{2+}}a_{SO_{4}^{2-}}a_{H_{2}O}^{2}\right)}_{eq}$$

where $a_{{Ca}^{2+}}$, $a_{SO_{4}^{2-}}$ and $a_{H_{2}O}$ are, respectively, the activities of the calcium and sulfate ions and water, with the ion activities $a_{i}$ expressed as the product of the activity coefficient $\gamma_{i}$ and the molality $m_{i}$ (in mol/kg) of species $i={Ca}^{2+},SO_{4}^{2-}$ and the subscript “eq” indicates that these characteristics are taken at the equilibrium composition. Following [30,31,32], $S_{g}$ was calculated by taking the extended Debye–Hückel model that included adjustable parameters to account for the variation in the water activity with the solution ionic strength:

(2) $$S_{g}=m_{{Ca}^{2+}}m_{SO_{4}^{2-}}\gamma_{{Ca}^{2+}}\gamma_{SO_{4}^{2-}}/K_{sp}^{0},$$

with ${log}_{10}\left(\gamma_{{Ca}^{2+}}\gamma_{SO_{4}^{2-}}\right)=-\left(z_{{Ca}^{2+}}^{2}+z_{SO_{4}^{2-}}^{2}\right)S_{DH}\sqrt{I}\left(1+A_{sp}\sqrt{I}\right)-B^{\prime }I+C^{\prime }I^{2}$, and $2I=z_{{Ca}^{2+}}^{2}m_{{Ca}^{2+}}+z_{SO_{4}^{2-}}^{2}m_{SO_{4}^{2-}}+z_{Na^{+}}m_{Na^{+}}+z_{{Cl}^{-}}m_{{Cl}^{-}}$, where I is the solution ionic strength in mol/kg, $K_{sp}^{0}$ is the solubility product constant for gypsum at zero ionic strength when $a_{H_{2}O}$ approaches unity, $z_{i}$ is the ion electrical charge, $S_{DH}$ is the Debye–Hückel coefficient, and $A_{sp}$, $B^{\prime }$, and $C^{\prime }$ are the adjustable parameters. The molal solubility of gypsum corresponds to $S_{g}=1$ with $m_{{Ca}^{2+}}=m_{SO_{4}^{2-}}$. The parameters in these expressions at 25°C in terms of mol/kg units are taken from [32]: ${log}_{10}K_{sp}^{0}=-4.374$, $S_{DH}=0.509$, $A_{sp}=1.500$, $B^{\prime }=0.0194$, $C^{\prime }=0.0134$.

### 2.3. Apparatuses and Procedures

#### 2.3.1. Stirred Reactor

The precipitation kinetics in a solution supersaturated with CaSO_4_ was studied at room temperature in a 1 L cylindrical vessel stirred with a VWR VOS 16 S41 Overhead Stirrer Mixer (VWR, Radnor, PA, USA) with a two-blade impeller having a diameter of 2.858 cm (1.125”) at a rotational speed of 427 rpm. The supersaturated mixture was prepared by charging the vessel with a 0.4 L solution of CaCl_2_/NaCl and a 0.4 L solution of Na_2_SO_4_/NaCl. Changes in the mixture’s electrical conductivity, turbidity, and Ca^2+^ concentration were measured with the relevant sensors submerged under the solution surface: a conductivity meter (CDH-SD1, Omega Engineering, Stamford, CT, USA), a turbidity probe (OBS-3+ Turbidity Sensor, Campbell Scientific, Logan, UT, USA, that measures turbidity in Formazin Nephelometric Unit (FNU) units according to ISO 7027 by using near-infrared light at 850 nm), and a Ca^2+^ ion selective electrode (perfectION™ Calcium Combination Electrode, Mettler-Toledo, Columbus, OH, USA, that measures the concentration of dissolved ions). Measurements were recorded by a data acquisition system (Labjack U6 Pro, Lakewood, CO, USA) at 5 Hz.

Once the experiment was completed, particles formed in the vessel were filtered out with a Pall Supor 200 Membrane Disc Filter with 0.2 µm pores and then dried overnight in a desiccator at room temperature.

#### 2.3.2. Flow Setup

Effects of the magnetic treatment of a solution supersaturated with CaSO_4_ on scale formation on an RO membrane were studied at room temperature in the flow setup with two branches A and B (Figure 1). A supersaturated solution in both branches was formed by feeding the pump through a T-junction pipe with solutions taken from two 1 L tanks; one filled with a solution of CaCl_2_/NaCl and the other with a solution of Na_2_SO_4_/NaCl (items 1 and 2 in Figure 1, respectively). Each branch of the setup included the following hardware: a laboratory cross-flow RO unit (Crossflow Cell CF042, Sterlitech, Kent, WA, USA; items 7 and 8 in Figure 1) with a flat membrane of 42 cm^2^ active area 9.208 cm (3.625”) × 4.572 cm (1.800”), slot depth 0.229 cm (0.09”) and maximum operating pressure and temperature of 68.9 bar (1000 psi) and 80 °C; a constant flow rate dual-head digital pump Lab Alliance Prep 100 (Scientific Systems, State College, PA, USA; items 5 and 6 in Figure 1) equipped with a pressure monitoring unit and a built-in pulse damper to deliver low flow rates with less than 2% RSD pulsations; and an OHAUS Scout Pro balance (OHAUS, Parsippany, NJ, USA) for measuring the weight of permeate collected in a glass beaker that was recorded with a data acquisition system (Logger Pro software 3.8.7, Vernier Technology, Beaverton, OR, USA) at a frequency of 60 Hz. The RO unit inlet was connected to the pump outlet with a thin metal tube. The 1 L tank was connected to the pump inlet with the Tygon S3 B-44-3 tubing, OD 0.476 cm (3/16”), ID 0.318 cm (1/8”) (Saint-Gobain Performance Plastics, Wayne, NJ, USA). The degree of solution supersaturation in the RO unit was computed from measurements of the solution electrical conductivity at the RO unit inlet and outlet. Retentate exits of both RO units were connected to an ultra-precision needle valve up to 206.8 bar (3000 psi), McMaster Carr, Robbinsville, NJ, USA), to simultaneously regulate pressure in both branches (item 10 in Figure 1). The pressure was monitored with a high-pressure Swagelok gauge 0–103.4 bar (0–1500 psi) with stainless tube and connectors, Mountainside, NJ, USA; item 9 in Figure 1.

**Figure 2:**
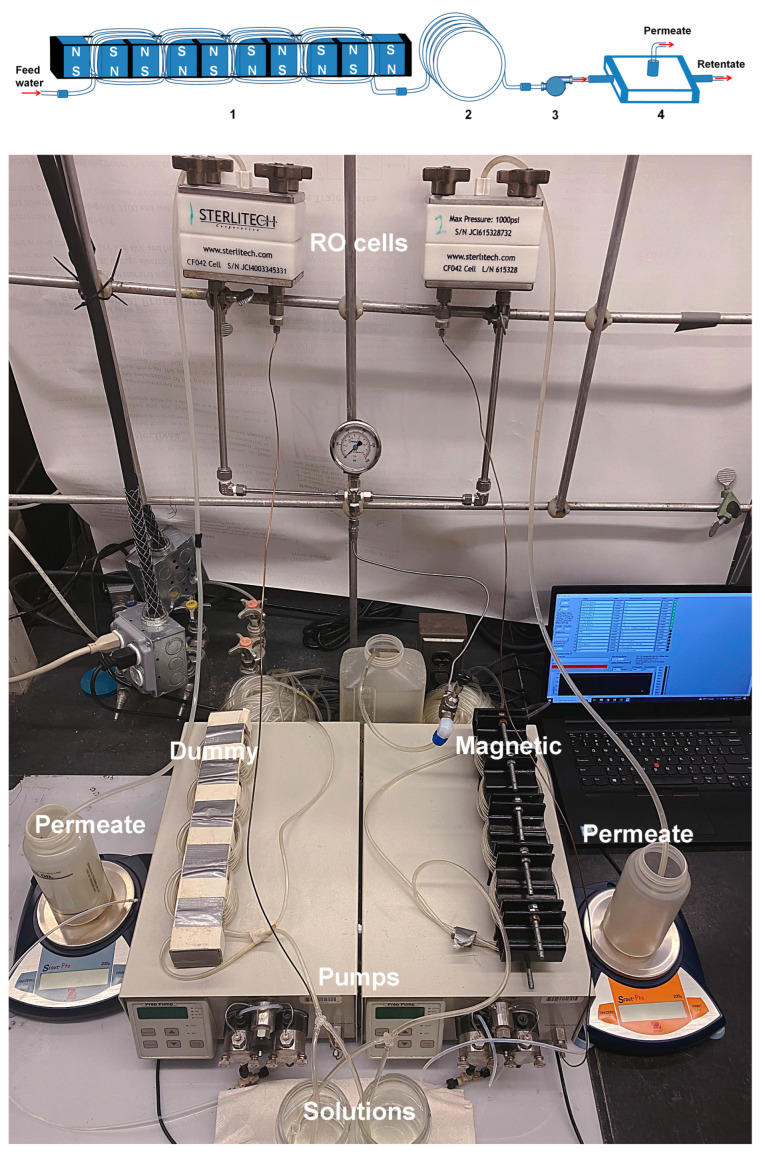
Top. Branch A [27]: 1, magnetic treatment unit with 24 coils measuring 610 cm (20 ft) tubing, h = 1 cm is the gap between magnets; 2, 3048 cm (100 ft) tubing; 3, constant flow rate pump; 4, RO unit. Bottom. Photo of the experimental setup.

Schematics of the setup branch A and the setup photo are presented in Figure 2. Branch A of the setup was equipped with five magnetic water softeners (Model MSW-4, Applied Magnets, Plano, TX, USA; item 3 in Figure 1 and item 1 in Figure 2, top) arranged in series. Each water softener was comprised of two parallel 5.08 cm (2”) × 2.54 cm (1”) × 2.54 cm (1”) neodymium magnets spaced at a distance of 1 cm apart using two stainless steel studs as guides. Approximately 610 cm (20 ft) of Tygon tubing was tightly wrapped around the magnets, forming 24 coils (Figure 2, bottom). The field strength of 0.8 T in the gap between magnets was measured with a DC Gaussmeter (Model 1-ST, AlphaLAB Inc., Salt Lake City, UT, USA). Branch B was equipped with a dummy unit (item 4 in Figure 1). The dummy unit was assembled from wooden blocks instead of magnets (Figure 2, bottom). Tygon tubing measuring 610 cm (20 ft) was also tightly wrapped around these wooden blocks, forming 24 coils. The tubing in each branch was connected to the pump inlet either directly or through an additional 3048 cm (100 ft) of tubing (item 2 in Figure 2, top). This additional tubing was used to explore the effect of magnetic memory in the RO unit by increasing water residence time in the flow assembly.

Experiments were conducted on flat-sheet polyamide membranes (Dow Filmtec BW30), which are used in large-scale RO industrial and municipal water treatment systems (Dow Water & Process Solutions, FILMTEC™ Reverse Osmosis Membranes, Edina, MN, USA). Rolls of membranes were stored in a container with DI water in a refrigerator. Specimens for flow experiments were prepared using a punch and die set provided by Sterlitech. They were rinsed with DI water, dried, and then mounted in RO units. DI water at a flow rate of 5–10 mL/min was used to clean the flow setup for at least 30 min before and after each experimental run. A supersaturated solution was introduced in the setup to replace water at a flow rate of 5 mL/min. As water was replaced, the solution flow rate was gradually increased to the desired value. Once the flow stabilized, the pressure in the setup was gradually increased to 55.2 bar (800 psi) by closing the needle valve (item 10 in Figure 1). Tests were carried out at an operating pressure greater than 41.4 bar (600 psi) recommended for BW30 membranes to raise the permeation flux and thereby enhance scale deposition. Tests were carried out for 5–25 min at fixed flow rates (Q = 10, 15, 20, and 25 mL/min) of a supersaturated solution. The collected amount of permeate was recorded. As the pump (items 5 and 6 in Figure 1) maintained a constant flow rate by increasing the pressure, the test was stopped earlier if the pump pressure accidentally exceeded 62.1 bar (900 psi) due to clogging.

The Reynolds numbers for the flow in the tubing and in the RO unit were respectively computed as ${Re}_{T}=u_{T}d_{T}/\nu$ and ${Re}_{RO}=u_{RO}h_{RO}/\nu$, where $u_{T}$ is the flow velocity in the tubing and $d_{T}$ is the tubing diameter; $u_{RO}$ is the flow velocity in the RO unit and $h_{RO}$ is the slot depth of the RO unit; and ν=1cSt is the water kinematic viscosity. The residence time in 610 cm (20 ft) coiled tubing arranged in the magnetic and dummy units (items 3 and 4 in Figure 1 and item 1 in Figure 2, top) was, respectively, computed as $t_{20ft}=L_{1}/u_{T}$ with $L_{1}$ = 610 cm (20 ft). The residence time in the 3048 cm (100 ft) coiled tubing (item 2 in Figure 2, top) was computed as $t_{100ft}=L_{2}/u_{T}$ with $L_{2}$= 3048 cm (100 ft). The total residence time in 3657 cm (120 ft) coiled tubing, when both coils were used, was $t_{120ft}=t_{100ft}+t_{20ft}$. The residence time in the RO unit was computed as $t_{RO}=L_{RO}/u_{RO}$ with $L_{RO}$ = 9.208 cm (3.625”). Unlike the flow in a straight tube, the fluid motion in a coiled tube is not parallel to the tube centerline. Centrifugal forces acting outward from the center of curvature of the tube centerline generate a secondary flow in the cross-sectional plane of the tube. The vortices arising in the coiled tubing are known to provide an effective means for mixing in a flowing fluid [30]. The intensity of the secondary flow is characterized by the Dean number [33] $De=Re\sqrt{d/2R}$ where d is the tube diameter and R is the radius of curvature of the tube centerline. The radii of curvature of 610 cm (20 ft) coiled tubing (items 3 and 4 in Figure 1 and item 1 in Figure 2, top) and 3048 cm (100 ft) coiled tubing (item 2 in Figure 2, top) were $R_{20ft}$= 3.61 cm (1.42”) and $R_{100ft}$= 7.01 cm (2.76”), respectively. All characteristics of the flow regimes listed above are summarized in Table 1.

Once a test was completed, the tested membrane was taken out of the RO units, gently dipped in DI water 10 times to remove loosely bound particles from the membrane that were formed near the membrane surface, and then dried for 1 day in a desiccator at room temperature. All tested membranes were stored dry in a desiccator for further analysis. For comparison, several tested membranes were dried in a desiccator without rinsing in DI water.

#### 2.3.3. Characterization Techniques

**Figure 3:**
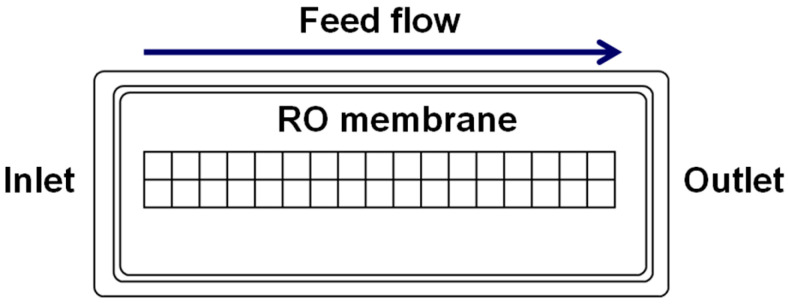
A grid of 5 mm × 5 mm squares drawn across the central part of the membrane surface; 17 in the flow direction and 2 in the perpendicular direction [27].

An optical microscope (Nikon SMZ1500, Melville, NY, USA) with 1× objective at 4× magnification was used to study the scale deposits formed on tested membranes. The objective was fitted with a polarizer and analyzer to improve the observation of the scale pattern. To measure the area of a membrane covered by scale deposits, a grid of 5 mm × 5 mm squares was drawn across the central part of the membrane surface, seventeen in the flow direction and two in the perpendicular direction (Figure 3). An image of each grid cell was recorded with a high-resolution CoolSNAP HQ 2 CCD camera (Photometrics, Tucson, AZ, USA). NIS Advanced Imaging software 4.40 (Nikon Instruments, Melville, NY, USA) and ImageJ 1.50 (the public domain image-processing software developed at the National Institute of Health, Bethesda, MD, USA) were used to compute the percentage of area covered with scale deposits in a grid cell.

A field emission scanning electron microscope (FESEM) LEO1530VP GEMINI (Carl Zeiss, Peabody, MA, USA) was used to observe the morphology of scale deposits formed on tested membranes. Specimens for scanning electron microscopy (SEM) analysis were cut out of a tested membrane and coated with carbon using a sputter coater (Bal-Tec MED 020 HR).

A PANalytical Empyrean Series 2 X-ray Diffractometer (Westborough, MA, USA) equipped with a Cu Kα X-ray source was used to determine the crystallinity of particles precipitated from a solution in the stirred reactor and the scale deposits formed on a tested membrane. An X-ray diffraction (XRD) pattern was recorded in the angular range (2θ) from 5° to 60° with a step-width of 0.013° at a scanning speed of 0.1° per second. The recorded diffraction patterns were compared with data on gypsum (CaSO_4_·2H_2_O) and anhydrite (CaSO_4_) in the instrument library.

Differential scanning calorimetry (DSC) curves of particles precipitated from a solution in the stirred reactor and collected from the scale deposits on a membrane in an RO unit were recorded on a Mettler Toledo DSC (Columbus, OH, USA). The calorimeter was calibrated with a Mettler Toledo indium sample. Measurements were conducted in semi-hermetically sealed aluminum cells on 3 mg specimens heated from room temperature to 250 °C at a rate of 10 °C/min.

#### 2.3.4. Statistical Analysis of Data

Under our experimental conditions (Figure 1), scale formation on the RO membrane surface from a solution supersaturated with CaSO_4_ could be affected by the following factors: magnetic treatment of a solution, the solution flow rate, the degree of solution supersaturation *ξ* in the RO unit, and the distance from the RO unit inlet to a particular location on the surface along the flow path. In the statistical analysis, measurements of scale deposits on tested membranes were arranged in k comparison groups, each affected by a specific factor or the level of this factor. A statistical hypothesis test [31], often referred to as the F-test, was then used to determine whether these factors or their levels affected scale formation. The null hypothesis, H_0_, was that a difference between measurements of the percentage of area covered with scale deposits in a grid cell on the membrane surface (Figure 3) within a particular group being compared with other groups appeared by chance. The alternative hypothesis, H_1_, was that this difference was influenced by factors under investigation. The test was based on a comparison between the means and variances among scale deposits computed separately for measurements within a specific group and the overall values computed for all measurements.

The following three-step procedure was used for testing the null hypothesis [34]: 

(**i**) Compute the mean ${\overset{-}{X}}_{j}$ and variance $S_{j}^{2}$ for each of k groups as

(3) $${\overset{-}{X}}_{j}=\frac{1}{n_{j}}\sum_{i=1}^{n_{j}}X_{ij}\;\mathrm{and}\;S_{j}^{2}=\frac{1}{n_{j}-1}\sum_{i=1}^{n_{j}}{\left(X_{ij}-{\overset{-}{X}}_{j}\right)}^{2},$$

where $n_{j}\left(j=1,\cdots ,k\right)$ is the number of measurements in the group j and $X_{ij}\left(i=1,\cdots ,n_{j}\right)$ are measurements in this group.

(**ii**) Compute the overall mean $\overset{-}{X}$ and variance $S^{2}$ for all the groups as

(4) $$\overset{-}{X}=\frac{1}{N}\sum_{j=1}^{k}\sum_{i=1}^{n_{j}}X_{ij}\;\mathrm{and}\;S^{2}=\frac{1}{N-1}\sum_{j=1}^{k}\sum_{i=1}^{n_{j}}{\left(X_{ij}-\overset{-}{X}\right)}^{2}$$

where $N=\sum_{j=1}^{k}n_{j}$ is total number of measurements; and (3) compute F factor as

(5) $$F={MS}_{B}/{MS}_{W}$$

with ${MS}_{B}=\frac{1}{k-1}\sum_{j=1}^{k}{n_{j}\left({\overset{-}{X}}_{j}-\overset{-}{X}\right)}^{2}$ and ${MS}_{W}=\frac{1}{N-k}\sum_{j=1}^{k}\sum_{i=1}^{n_{j}}{\left(X_{ij}-{\overset{-}{X}}_{j}\right)}^{2}$, where ${MS}_{B}$ characterizes the mean variability between the groups and ${MS}_{W}$ characterizes the mean variability within these groups. The value of F in Equation (5) will be large only if the variability between the groups is large compared to the variability within the groups. There are two criteria for rejecting or accepting the null hypothesis. Both depend on two degrees of freedom, k−1 and N−k, and the chosen significance level α that yields the confidence level $100\cdot \left(1-\alpha \right)\%$ [34]. One of them is to calculate $F_{\alpha }$ that is a function of α and the degrees of freedom: F should exceed $F_{\alpha }$ for the null hypothesis to be rejected. The other is to calculate the p-value that is a function of F and the degrees of freedom and find α for rejecting, α>p, or accepting, α≤p, the null hypothesis.

## 3. Results and Discussion

### 3.1. Precipitation Kinetics

**Figure 4:**
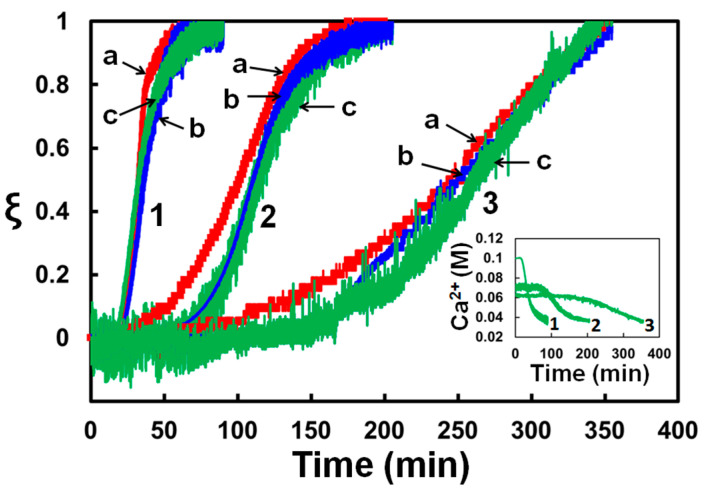
Variation of the relative change in the solution (a) electrical conductivity, (b) turbidity, and (c) Ca^2+^ concentration during calcium sulfate precipitation for solutions with (1) 100, (2) 70, and (3) 60 mM of CaSO_4_ and 500 mM of NaCl. Inset: Variation in Ca^2+^ concentration with time [27].

Changes in the electrical conductivity, turbidity, and ${\mathrm{Ca}}^{2+}$ concentration during calcium sulfate precipitation in the stirred reactor were monitored at room temperature in three solutions with 100, 70, and 60 mM of CaSO_4_ and 0.5 M of NaCl. They were prepared by mixing stock solutions with *x* and *y* in Equation (1) equal to 0.15, 0.1; 0.18, 0.07; and 0.19, 0.06, respectively. Relative changes of each of these characteristics, ξ, with time are plotted in Figure 4; ξ=0 at the beginning and ξ=1 at the end of precipitation. As can be seen in Figure 4, the relative variations of these properties are similar, so each of them can be used to characterize the degree of the solution supersaturation. As measuring the electrical conductivity is easier to implement in a flow system, the degree of solution supersaturation in the RO unit was evaluated by measuring its electrical conductivity at the unit inlet and outlet. Specifically, the value of ξ in flow experiments was computed as the relative change in the solution conductivity in comparison with the total change, from 54.2±1.5mS/cm to 46.8±1.3mS/cm, measured in the stirred reactor.

Precipitation from a supersaturated solution begins with the appearance of nuclei that, once formed, grow into larger particles. The induction periods of precipitation for these solutions, taken as the time when ξ underwent a sudden increase from zero in Figure 4, are 20 ± 2, 62 ± 6, and 130 ± 14 min. The concentrations of ${Ca}^{2+}$ ions at the end of precipitation shown in Figure 4 are 37.5 ± 1.2, 36.6 ± 0.7, and 36.2 ± 0.4 mM, respectively. These measurements compare well with data on the equilibrium solubility of gypsum CaSO_4_·2H_2_O in aqueous solutions of NaCl [30,31,32].

The solubility of gypsum in a 0.5 M NaCl solution computed from Equation (2) at $S_{g}=1$ is 36.16 mM, which agrees well with our measurements. The values of the supersaturation ratio $S_{g}$ calculated from Equation (2) for the three supersaturated solutions used in our experiments are, respectively, 5.92, 3.23, and 2.47. In terms of classical nucleation theory, the dependence of the induction time for the nucleation of gypsum particles on $S_{g}$ is given by [35]

(6) $${log}_{10}t_{ind}=A+B/{log}_{10}^{2}S_{g}\;\mathrm{with}\;B=\beta \sigma^{3}V_{m}^{2}N_{A}f/n^{2}{\left(2.3R_{g}T\right)}^{3},$$

where A depends on the frequencies with which ions ${Ca}^{2+}$ and $SO_{4}^{2-}$ in the solution attach and detach the gypsum nucleus and B represents a thermodynamic barrier to form a nucleus, β is the shape factor equal to 16π/3 for a spherical nucleus, σ is the gypsum/solution interfacial energy, $V_{m}=74.44{cm}^{3}/g$ is the gypsum molar volume, $N_{A}$ is the Avogadro number, $R_{g}$ is the gas constant, T is the absolute temperature, n=2 is the number of ions in which CaSO_4_·2H_2_O dissociates, and f is the factor that equals 1 for homogenous nucleation when a gypsum nucleus forms spontaneously in the solution and characterizes a decrease of interfacial energy for heterogeneous nucleation when it forms on the surface of a foreign solid particle. The slope of the straight line given by Equation (6) can be used to evaluate the gypsum/solution interfacial energy [36]. The regime for values of $S_{g}$ greater than 2 is usually attributed to homogenous nucleation with spherical nuclei. Using our data, we obtain $\sigma \approx 42\;mJ/m^{2}$ for room temperature that lies within the range $8–44\;mJ/m^{2}$ of published data [37,38,39,40,41,42,43,44,45] and agrees well with the range $37–44\;mJ/m^{2}$ calculated from measurements in a stirred reactor [37,38,39,40].

The presented measurements demonstrate that ξ computed from changes in the electrical conductivity, turbidity, and ${Ca}^{2+}$ concentration provides a reliable parameter to characterize a supersaturated solution flowing through an RO unit.

### 3.2. Scale Formation in RO Unit

The formation of calcium sulfate scale on RO membranes was studied at room temperature for a solution with 70 mM of CaSO_4_ and 0.5 M of NaCl. Experiments were carried out in the setup depicted in Figure 1 with and without the additional 3048 cm (100 ft) coiled tubing shown in Figure 2. Two membranes were tested in every run. A membrane in RO unit 7 (Figure 1) was exposed to the solution subjected to the magnetic field, whereas the other membrane in RO unit 8 (Figure 1) was exposed to the solution not treated with the field. Runs were carried out with and without the 3048 cm (100 ft) coiled tubing shown in Figure 2. All membranes were washed after the test. The area of a membrane covered with scale deposits was calculated on the grid cells (Figure 3). Differences between data on electrical conductivity at the RO unit inlet of solutions flowing through the magnetic and dummy units, 56.1±0.6 mS/cm, and the initial solution conductivity, 54.2±1.5 mS/cm, were lying within the measurement errors that corresponded to ξ=0. The same value, 56.1±0.7 mS/cm, was found for the solution conductivity at the RO unit outlet in both branches of the setup without the 100 ft coiled tubing. When the 3048 cm (100 ft) coiled tubing was placed in both setup branches, the solution conductivity at the RO unit outlet was measured to be 53.9±0.8 mS/cm for all flow rates that yielded ξ=0.14.

The variation in the permeate weight with time for tested membranes was found to be well approximated as

(7) $$q\left(g\right)=-a{t\left(min\right)}^{2}+bt\left(min\right)$$

with the coefficient of determination $R^{2}>0.995$ for all tests. The negative first term in Equation (7) describes a decrease in the permeate flow rate due to scale formation. Table 2 summarizes the results of the statistical analysis of the mean and variance of coefficients in Equation (7) obtained in both setup branches with and without the presence of 3048 cm (100 ft) coiled tubing at different feed flow rates. The parameters in Table 2 were calculated with the use of Equations (3)–(5). The statistical significance of different factors listed in Table 2 was evaluated for α=0.05 corresponding to a confidence level of 95%. As can be seen in Table 2, the values of F computed for effects of the magnetic treatment and the tubing length (magnetic memory) on a and b in Equation (7) are smaller than $F_{0.05}=4.05$; α=0.05 is also smaller than the p-value. It means that their influence on the permeate flow rate is statistically insignificant with a confidence level of 95%. The influence of the feed flow rate on coefficient b in Equation (7) is also statistically insignificant. However, the influence of the flow rate on coefficient a in Equation (7) appears to be statistically significant as $F=5.83>F_{0.05}=2.88$ and p=0.03 is smaller than α=0.05 (Table 2). Specifically, a decrease in the feed flow rate raises a.

**Figure 5:**
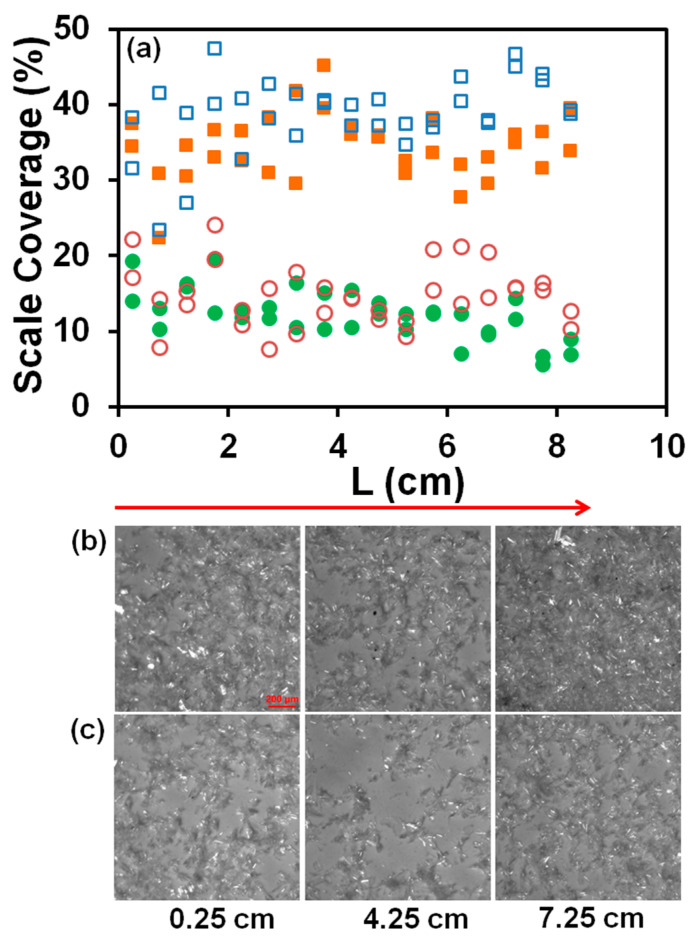
Scale deposits formed in grid cells shown in Figure 3. L (cm) is measured along the flow path from the RO unit inlet. Test was conducted at a flow rate of 20 mL/min for 10 min in 70 mM CaSO_4_, 0.5 M NaCl solution. The magnetic and dummy units were connected to the pump inlet with additional 3048 cm (100 ft) tubing. (**a**) The percentage of the membrane area covered with scale deposits. Each point is the average taken over two cells in the direction perpendicular to the flow. Unfilled and filled symbols represent data for magnetic and dummy units; squares and circles represent data for not washed and washed membranes. (**b**,**c**) Photos of the membrane surface in three grid cells taken at the distances of 0.25 cm, 4.25, and 7.25 cm from the RO unit inlet: (**b**) not washed membranes; (**c**) washed membranes. The scale bar of 200 μm is the same in all photos [27].

Scale deposits on membranes under our experimental conditions were formed directly by surface crystallization and by gravity settling of particles precipitated in the bulk, as the calcium sulfate density is about 2.3 g/cm^3^. Dipping the tested membranes in DI water ten times removed most of the loosely adherent particles that settled from the bulk. As an example, photos of washed and not washed membranes tested at a flow rate of 20 mL/min for 10 min in solutions flowing through the magnetic and dummy units are shown in Figure 5. The magnetic and dummy units were connected to the pump inlet with an additional 3048 cm (100 ft) of tubing (Figure 2). The percentage of the membrane area covered with scale deposits was measured in each cell of the grid shown in Figure 3. Variations of the scale coverage averaged over two cells in the perpendicular direction are plotted in Figure 5 along the flow path. As can be seen in Figure 5, washing reduced the deposit coverage of the membranes by about 20%.

The percentage of the scale coverage averaged over the entire grid was used to evaluate the effects of the magnetic treatment, the presence of 3048 cm (100 ft) coiled tubing (magnetic memory), and the feed flow rate on scale formation. Table 3 summarizes the results of the statistical analysis of measurements in 48 runs, each lasting about 15 min. Parameters in Table 3 were computed from Equations (3)–(5). The value α=0.05 for a confidence level of 95% was taken to evaluate the statistical significance of the considered factors.

**Figure 6:**
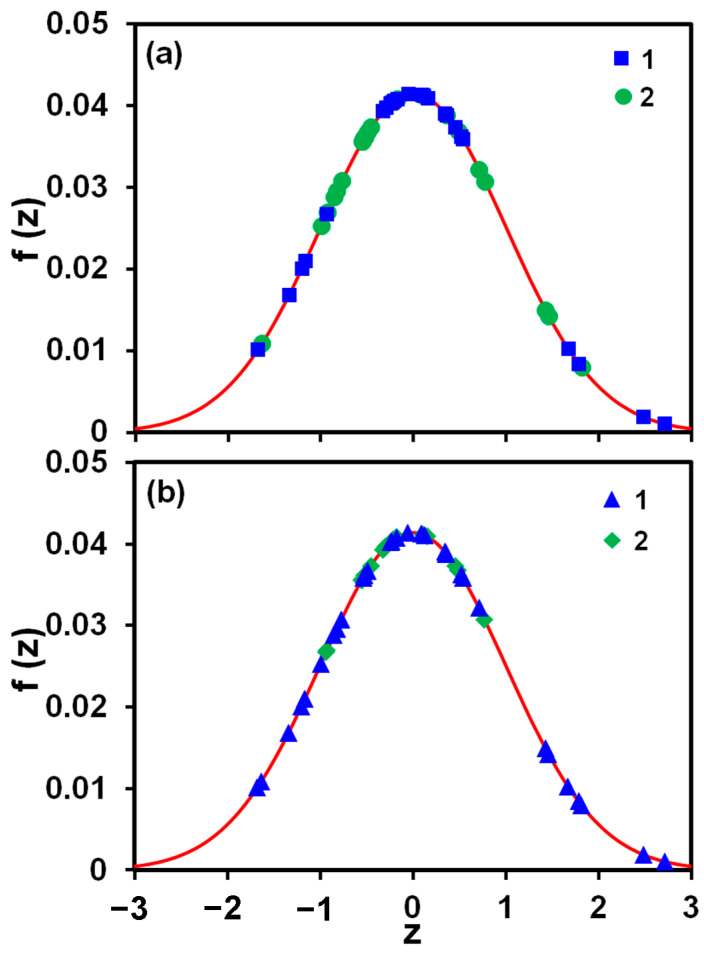
The Gaussian distribution, Equation (8), and the histogram of the percentage of the membrane area covered with scale deposits, both normalized with the mean and variance taken from Table 3 for all tests, the width of the histogram bin is 0.5: (**a**) data with, 1, and without, 2, magnetic treatment; (**b**) data with, 1, and without, 2, the presence of 3048 cm (100 ft) coiled tubing [27].

As can be seen in Table 3, the values of F computed for the effects of the magnetic treatment and the tubing length (magnetic memory) on the membrane area covered with scale deposits are smaller than $F_{0.05}=4.05$; α=0.05 is also smaller than the p-value. It means that their influence on the scale formation rate is statistically insignificant with a confidence level of 95%. This result is illustrated by plots in Figure 6, which show that measurements of the percentage of the scale coverage with/without magnetic treatment and with/without the presence of 3048 cm (100 ft) coiled tubing follow the Gaussian distribution, $f\left(z\right)$, with the mean and variance taken from Table 3 for all tests:

(8) $$f\left(z\right)=\frac{exp\left(-z^{2}/2\right)}{S\sqrt{2\pi }},\;\mathrm{with}\;z=\frac{X-\overset{-}{X}}{S}$$

On the other hand, the influence of the feed flow rate on scale formation appears to be statistically significant since $F=3.53>F_{0.05}=2.88$ and p=0.025 is smaller than α=0.05 (Table 3). The observation that reducing the feed flow rate intensifies scale formation, as Table 3 demonstrates, is consistent with the data in Table 2 on increasing the coefficient magnitude of a in Equation (7) with decreasing the feed flow rate. Facilitating scale formation by decreasing the feed flow rate can be related to reducing the shear stress exerted on deposits crystallized on a membrane.

**Figure 7:**
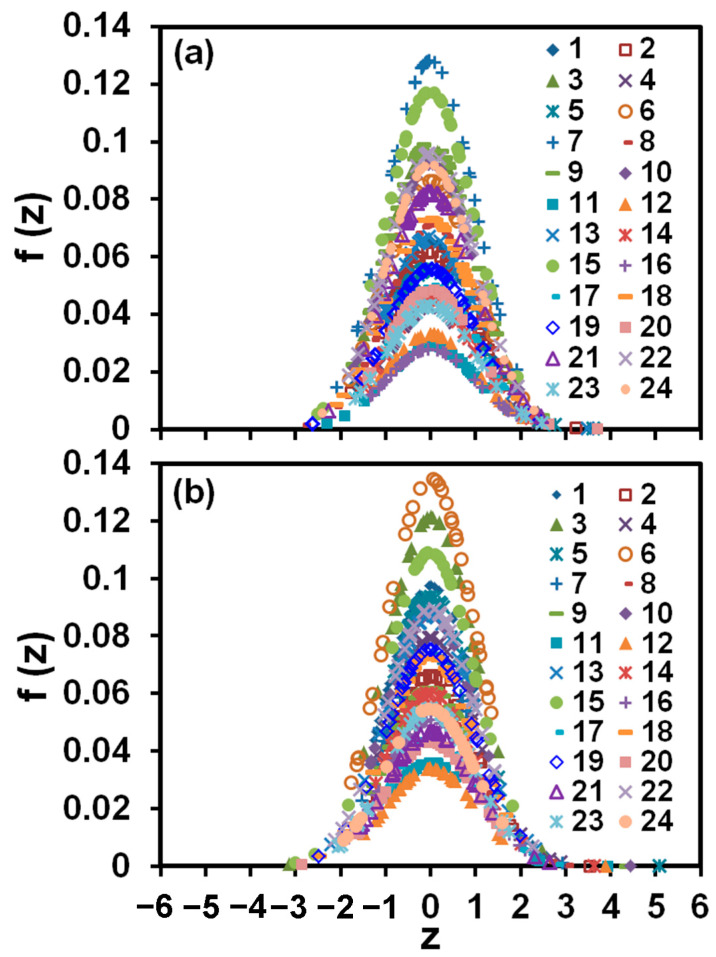
In the histogram of the percentage of the scale coverage of each grid cell normalized with the mean and variance taken over the entire grid (Figure 3), the width of the histogram bin is 0.5. Measurements on 24 membranes for solutions (**a**) exposed and (**b**) not exposed to the magnetic field [27].

Plots presented in Figure 7 demonstrate that measurements of the percentage of scale coverage of each grid cell on the membrane, normalized with the mean and variance taken over the entire grid, follow the Gaussian distribution. Moreover, most measurements fall within one standard deviation of the mean. These observations show clearly that scale formation was not affected by the distance of a particular location on the membrane surface from the RO unit inlet and, therefore, was not controlled by the diffusion of salt ions towards the membrane surface. This feature of our setup allows, for the first time, the detailed study of scale formation in the regime controlled by surface crystallization that is typical of industrial RO systems, which operate at a high degree of concentration polarization in the vicinity of the membrane surface [28].

**Figure 8:**
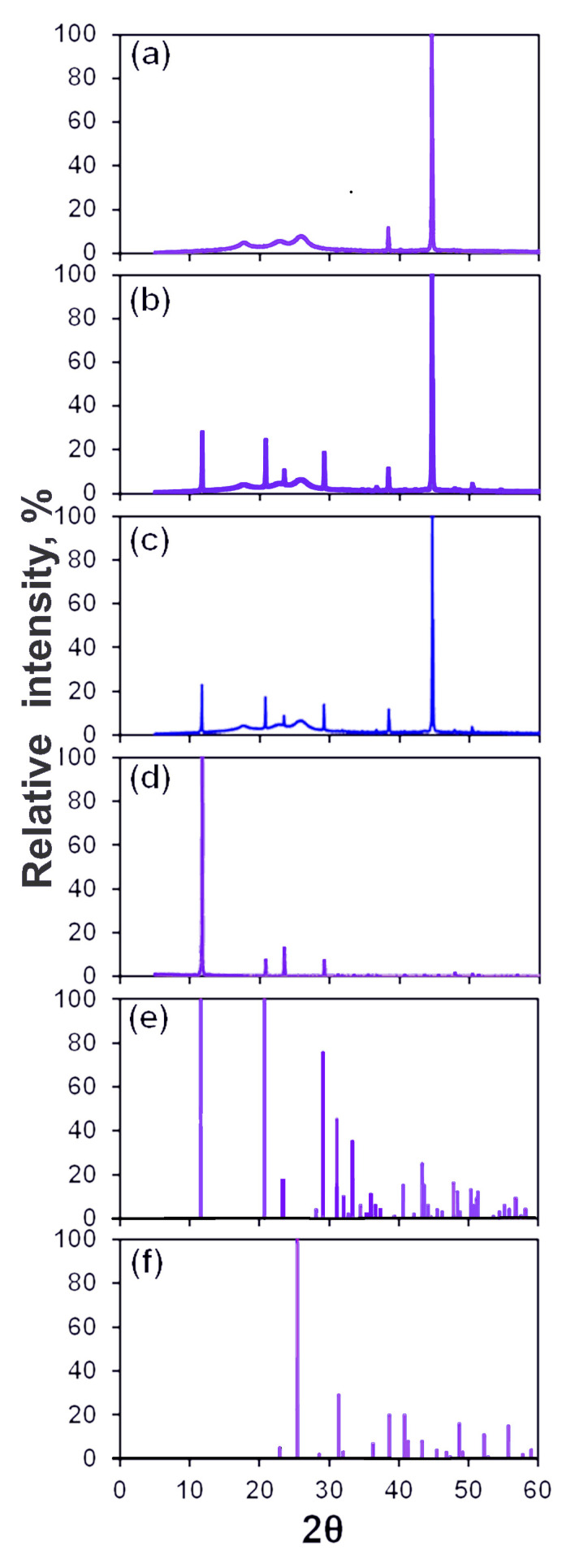
X-ray diffraction patterns: (**a**) A fresh membrane; (**b**,**c**) washed membranes exposed in the flow setup to 70 mM CaSO_4_/0.5 M NaCl solution pumped at a flow rate of 20 mL/min for 15 min through (**b**) dummy and (**c**) magnetic treatment units; (**d**) particles precipitated from 70 mM CaSO_4_/0.5 M NaCl solution in the stirred reactor; (**e**,**f**) patterns from the instrument library of (**e**) dihydrate (CaSO_4_·2H_2_O), No. 00-033-0311, and (**f**) anhydrite (CaSO_4_), No. 00-037-1496, forms of calcium sulfate [27].

The X-ray diffraction patterns obtained from washed membranes taken from dummy and magnetic treatment branches are shown in Figure 8. These membranes were tested at a flow rate of 20 mL/min for 15 min. Diffraction patterns obtained from a fresh membrane and from particles collected in the stirred reactor and patterns in the instrument library of calcium sulfate dihydrate (CaSO_4_·2H_2_O) and anhydrite (CaSO_4_) are also presented in Figure 8. Two XRD peaks in the pattern of the fresh membrane in Figure 8a characterize the membrane crystallinity. They also appear in the patterns of tested membranes in Figure 8b,c. The patterns in Figure 8b,c that were obtained from membranes taken from dummy and magnetic treatment branches are practically identical. Four of the leftmost XRD peaks in these patterns coincide with four peaks in the pattern in Figure 8d obtained from particles collected in the stirred reactor. These are the major diffraction peaks (020), (021), (130), and (041) of calcium sulfate dihydrate with positions (2θ) at 11.61°, 20.71°, 23.41° and 29.11, respectively [46]. The Miller indices of these peaks are referred to in the instrument library pattern No. 00-033-0311 in Figure 8e. As can be seen in Figure 8f, there are no peaks of calcium sulfate anhydrite on patterns obtained from membranes. Relationships between intensities of the gypsum XRD peaks are known to strongly depend on the conditions under which these crystals are formed [46]. The weak gypsum peak of (130) face and comparable intensities of other peaks in Figure 8b,c indicate that needle-like microcrystals mainly form on the membrane surface [46]. The fact that the peak of (020) dominates Figure 8d indicates the formation of granular microcrystals in the stirred reactor.

**Figure 9:**
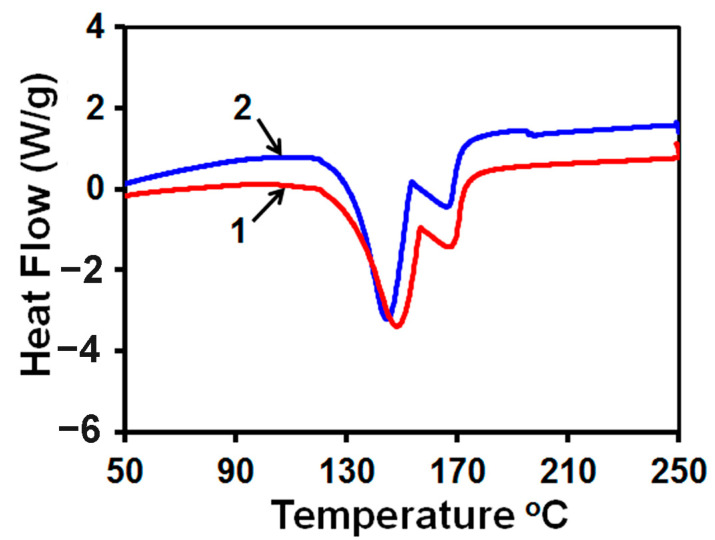
Overlay of DSC curves obtained from particles (1) precipitated from 70 mM CaSO_4_/0.5 M NaCl solution in the stirred reactor and (2) collected from scale deposits on an RO membrane exposed to the flow of 70 mM CaSO_4_/0.5 M NaCl solution at a flow rate of 20 mL/min for 15 min. The solution was treated with a magnetic field [27].

The DSC curves shown in Figure 9 were obtained from gypsum particles collected in the stirred reactor and from scale deposits on the tested RO membrane. Both curves demonstrate two endothermic peaks in the 130–180 °C range that are related to the two-step process of gypsum dehydration [47,48]. A slight difference between these curves can be attributed to the different morphologies of particles formed in the reactor and on the RO membrane.

### 3.3. Morphology of Scale Deposits

**Figure 10:**
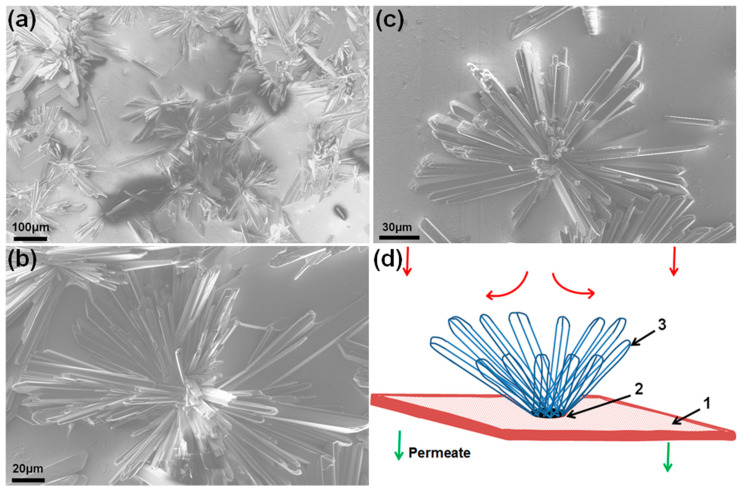
Rosette structures of scale deposits: (**a**–**c**) SEM micrographs of scale deposits formed on a membrane exposed to a flow of 70 mM CaSO_4_/0.5 M NaCl solution at a flow rate of 20 mL/min for 15 min. The solution was treated with the magnetic field; (**d**) formation of the rosette structure: 1, membrane surface; 2, gypsum surface nucleus; 3, growing needle crystals; arrows show flow towards the membrane surface [27].

Scale deposits on the membrane surface exhibit a rosette structure formed by needle-like crystals growing radially from a single growth center crystallized on the membrane surface (Figure 10). It is similar to the morphology of gypsum deposits on RO membranes observed in [49,50,51]. Those experiments were conducted at low supersaturation of the feed solution when scale formation was governed by the diffusion of salt ions towards the membrane. Accordingly, crystals in [49,50,51] were found to grow in the direction opposite to the feed flow by forming a well-developed rosette morphology at the channel exit. In our experiments carried out at high supersaturation of the feed solution, rosette structures formed all over the membrane surface (Figure 5). This fact, taken together with observations for RO [49,50,51] and nanofiltration [52] membranes, indicates that the formation of gypsum rosette structures is controlled by the precipitation kinetics in the vicinity of the membrane surface. The schematic in Figure 10d illustrates the mechanism of the positive feedback that causes the self-accelerated growth of rosette structures on the membrane surface. The appearance of an impermeable scale deposit on the membrane surface rearranges the direction of the solution flow towards the membrane (Figure 10d). It leads to an increase in the local concentration of salt ions at the periphery of the scale deposit, thereby facilitating the outward-oriented growth of gypsum needles. That, in turn, expands the area of the membrane surface impermeable for water to continue redistributing the flow and thereby raises the concentration of salt ions at the needle ends to accelerate their growth.

Refs. [49,50,51,52,53] attributed the formation of gypsum rosette structures on membranes to surface crystallization. However, a similar morphology, referred to as star- or sea urchin-shaped aggregates, could also form in the bulk of a supersaturated solution. Observations of similar structures in evaporating droplets of the solution of a calcium salt and sulfuric acid date back to the beginning of the last century [54]. In recent studies, this morphology was found to form under certain conditions of the bulk precipitation of calcium sulfate from supersaturated aqueous solutions of anhydrous CaSO_4_ [55], calcium hydroxide/sulfuric acid with added citric acid [56], calcium sulfate hemihydrate (CaSO_4_·0.5H_2_O) with added citric acid [57], and CaCl_2_/Na_2_SO_4_ [58]. Gypsum was demonstrated to form through non-classical nucleation pathways via amorphous calcium sulfate and calcium sulfate hemihydrate intermediates [59,60,61,62,63]. On the contrary, the classical mechanism implies that the critical nucleus core has the crystalline structure of a solid crystallized from a solution [29]. The multistep pathway of gypsum precipitation that is accompanied by the reorganization of intermediates is considered to be the main factor affecting gypsum morphology. Understanding gypsum precipitation in RO processes will lead to more efficient anti-scaling strategies. The presented methodology of testing under a controlled level of supersaturation can be used to explore the effect of the solution supersaturation on the pathways of gypsum surface crystallization on RO membranes. The outcome of these experiments will help find a way to reduce membrane scaling.

## 4. Conclusions

The paper presents a methodology [27] for using short-term tests to evaluate the effectiveness of magnetic water treatment for reducing mineral scaling in reverse osmosis (RO). Testing was conducted in the transient flow regime of a single-pass flow through an RO unit. Experiments were carried out on an aqueous CaSO_4_/NaCl solution at a controlled level of supersaturation with CaSO_4_. The feed solution was formed by mixing CaCl_2_/NaCl and Na_2_SO_4_/NaCl solutions. To quantify the solution supersaturation, variations in the electrical conductivity, turbidity, and Ca^2+^ concentration during gypsum precipitation were measured in the stirred reactor. As the relative changes of these properties were found to be similar, measuring the solution’s electrical conductivity was chosen to characterize the supersaturation level in the flow system. To improve the test resolution, the flow setup consisted of two similar branches, both equipped with an RO unit and a constant flow rate pump. These branches operated in parallel at the same flow rate and transmembrane pressure and were fed with a solution at the same level of supersaturation, but the solution was exposed to a magnetic field only in one of them. The described methodology allowed, for the first time, the detailed study of magnetic treatment on scale formation under controlled solution supersaturation. The effects of the magnetic treatment of the feed solution on gypsum scaling under our experimental conditions were found to be statistically insignificant with a confidence level of 95%.

The presented experimental results should not be considered to negate the potential efficiency of the treatment of feed water with a magnetic field in specific applications. As stated in the Introduction, the purpose of the methodology [27] is to use benchtop tests for the evaluation of the benefits of magnetic water treatment in a large industrial RO system. The approach is to measure the water’s electrical conductivity along the flow path through the system of interest and then determine local water supersaturation from data on changes in water electrical conductivity in a stirred reactor. The magnetic water treatment would be useful only if benchtop tests revealed its efficiency over the range of local water supersaturation observed in the system of interest. The methodology [27] can also be useful for evaluating the efficiency of other technologies for water treatment in industrial flow systems, even for testing the efficacy of specific antiscalant additives. The key is to determine the local water supersaturation along the flow path in the system of interest. The outcome of laboratory tests would then reveal the range of salt supersaturation for which the treatment technology is effective.

## 5. Patents

Results of the reported experiments were used to develop patent [27].

## Figures and Tables

**Table 1:** Characteristics of flow regimes used in conducted tests [27].

Q (mL/min)	$u_{T}$ (cm/s)	$u_{RO}$ (cm/s)	${Re}_{T}$	${Re}_{RO}$	${De}_{20ft}$	${De}_{100ft}$	$t_{20ft}$ (min)	$t_{100ft}$ (min)	$t_{120ft}$ (min)	$t_{RO}$ (s)
10	2.1	0.16	67	3.6	14.0	10.1	4.8	24.1	29.0	57.7
15	3.2	0.24	100	5.5	21.0	15.1	3.2	16.1	19.3	38.5
20	4.2	0.32	134	7.3	28.0	20.1	2.4	12.1	14.5	28.9
25	5.3	0.40	167	9.1	35.0	25.2	1.9	9.7	11.6	23.1

**Table 2:** Data on coefficients in Equation (7) for permeate weight [27].

All tested membranes	N	$\overset{-}{a}$, g/min^2^	$S_{a}$, g/min^2^	$\overset{-}{b}$, g/min	$S_{b}$, g/min
	48	0.0221	0.0005	1.115	0.147
**Effects of magnetic treatment**					
Groups	$n_{j}$	${\overset{-}{a}}_{j}$, g/min^2^	$S_{a_{j}}$, g/min^2^	${\overset{-}{b}}_{j}$, g/min	$S_{b_{j}}$, g/min
j=1 exposed to magnetic field	24	0.0272	0.0005	1.123	0.159
j=2 not exposed to magnetic field	24	0.0212	0.0002	1.107	0.142
F-test	F	$F_{\alpha },\alpha =0.05$		p	
Coefficient a	1.56	4.05		0.22	
Coefficient b	0.02	4.05		0.89	
**Effects of additional 3048 cm (100 ft)**					
Groups	$n_{j}$	${\overset{-}{a}}_{j}$, g/min^2^	$S_{a_{j}}$, g/min^2^	${\overset{-}{b}}_{j}$, g/min	$S_{b_{j}}$, g/min
j=1, without 100 ft coiled tubing	16	0.0156	0.0003	1.047	0.184
j=2, with 100 ft coiled tubing	32	0.0253	0.0005	1.149	0.130
F-test	F	$F_{\alpha },\alpha =0.05$		p	
Coefficient a	2.22	4.05		0.14	
Coefficient b	0.74	4.05		0.39	
**Effects of feed flow rates**					
Groups	$n_{j}$	${\overset{-}{a}}_{j}$, g/min^2^	$S_{a_{j}}$, g/min^2^	${\overset{-}{b}}_{j}$, g/min	$S_{b_{j}}$, g/min
j=1, Q = 10 mL/min	14	0.0416	0.0003	1.228	0.149
j=2, Q = 15 mL/min	8	0.0250	0.0003	1.287	0.165
j=3, Q = 20 mL/min	10	0.0154	0.0003	0.962	0.156
j=4 Q = 25 mL/min	6	0.0163	4 × 10^−6^	0.873	0.064
F-test	F	$F_{\alpha },\alpha =0.05$		p	
Coefficient a	5.83	2.88		0.003	
Coefficient b	2.35	2.88		0.089	

**Table 3:** Data on the membrane area covered with scale deposits [27].

All tested membranes	N	$\overset{-}{X}$, %	S, %
	48	23.45	9.63
Groups	$n_{j}$	${\overset{-}{X}}_{j}$, %	$S_{j}$, %
**Effects of magnetic treatment of solution**			
j=1, exposed to magnetic field	24	24.81	8.34
j=2, not exposed to magnetic field	24	22.09	10.78
F-test	F	$F_{\alpha },\alpha =0.05$	p
	0.95	4.05	0.33
**Effects of additional 3048 cm (100 ft) coiled tubing**			
j=1, without 100 ft coiled tubing	16	21.54	4.71
j=2, with 100 ft coiled tubing	32	24.40	11.28
F-test	F	$F_{\alpha },\alpha =0.05$	p
	0.94	4.05	0.34
**Effects of feed flow rates**			
j=1, Q = 10 mL/min	14	30.83	113.44
j=2, Q = 15 mL/min	8	20.50	32.94
j=3, Q = 20 mL/min	10	21.12	108.29
j=4, Q = 25 mL/min	6	21.40	16.11
F-test	F	$F_{\alpha },\alpha =0.05$	p
	3.53	2.88	0.025

## Data Availability

The data presented in this study are available on request from the corresponding author.
